# Mutations in Wnt2 Alter Presynaptic Motor Neuron Morphology and Presynaptic Protein Localization at the *Drosophila* Neuromuscular Junction

**DOI:** 10.1371/journal.pone.0012778

**Published:** 2010-09-15

**Authors:** Faith L. W. Liebl, Cassandra McKeown, Ying Yao, Huey K. Hing

**Affiliations:** 1 Department of Biological Sciences, Southern Illinois University Edwardsville, Edwardsville, Illinois, United States of America; 2 Department of Biology, University of Washington, Seattle, Washington, United States of America; 3 Department of Biology, State University of New York Brockport, Brockport, New York, United States of America; Institut de la Vision, France

## Abstract

Wnt proteins are secreted proteins involved in a number of developmental processes including neural development and synaptogenesis. We sought to determine the role of the *Drosophila* Wnt7b ortholog, Wnt2, using the neuromuscular junction (NMJ). Mutations in *wnt2* produce an increase in the number of presynaptic branches and a reduction in immunolabeling of the active zone proteins, Bruchpilot and synaptobrevin, at the NMJ. There was no change, however, in immunolabeling for the presynaptic proteins cysteine-string protein (CSP) and synaptotagmin, nor the postsynaptic proteins GluRIIA and DLG at the NMJ. Consistent with the presynaptic defects, *wnt2* mutants exhibit approximately a 50% reduction in evoked excitatory junctional currents. Rescue, RNAi, and tissue-specific qRT-PCR experiments indicate that Wnt2 is expressed by the postsynaptic cell where it may serve as a retrograde signal that regulates presynaptic morphology and the localization of presynaptic proteins.

## Introduction

Synapses are specialized structures that allow neurons to communicate with one another. This communication is achieved by converting the electrical signal of the axon into a chemical signal at structures called active zones (AZs). AZs are made up of a dense protein matrix that collectively participates in synaptic vesicle exocytosis (for reviews see [1], [2]). Important components of this matrix include Ca^2+^ channels [3], proteins involved in vesicle fusion including SNAP-25, Synaptobrevin, and Syntaxin [4], [5], scaffolding proteins including Bassoon [6], Piccolo [7], CAST/ELKS/Bruchpilot [8], [9], [10], cell adhesion molecules including cadherins [11] and neuroligins [12], [13]. The assembly of AZs is thought to occur quickly after axonal target recognition and contact with the postsynaptic cell. AZ proteins are packaged and transported in vesicles and delivered to synaptic locations [14], [15], [16]. The mechanisms by which AZs are properly localized and maintained are largely unknown but likely involve transsynaptic signaling to coordinate the development of the presynaptic neuron with the postsynaptic cell.

The Wnt family of secreted glycoproteins, well characterized for their roles in several developmental processes including cell fate specification, axis patterning, and neural development (for reviews see [17]–[19]), also regulate synapse development (for reviews see [20], [21]). For example, mouse Wnt7a regulates the organization of presynaptic microtubules and clustering of the presynaptic proteins synapsin I [22] and synaptophysin [22], [23]. Similarly, the mouse Wnt3 protein increases axon branching, growth cone size, and synapsin I clustering in presynaptic sensory neurons [24]. Wnts 3, 5a, 7a, and 7b are expressed in the hippocampus along with Frizzled receptors where these proteins regulate synapse formation [25]. In *Drosophila*, Wingless (Wg) governs the development of both pre- and postsynaptic structures [26]–[28]. Finally, *Drosophila* Wnt5 positively regulates neuromuscular junction (NMJ) growth and the synaptic localization of active zone proteins [29].

Wnt proteins signal through at least three types of receptors including the Frizzled (Fz) family of receptors [18], [19], the Ryk/Drl family of receptor tyrosine kinases [30]–[34], and the receptor tyrosine kinase-like orphan receptor (Ror) [35], [36]. *Drosophila* Wnt5 has been shown to signal via Drl to mediate events such as axon repulsion [34], olfactory map formation [37], [38], and NMJ development [29]. In mammals, Wnt5a signals through Ror2 to regulate morphogenesis [35], [36] and members of the Fz family [39], [40] to promote cytoskeletal remodeling.

The *Drosophila* larval NMJ is a well-established model system for dissecting the molecular basis of synapse formation, growth, and remodeling. These synapses are similar to mammalian central synapses in that they are glutamatergic and remodel in response to activity [41], [42]. Using this system, we show *in vivo* for the first time that the *Drosophila wnt7b* ortholog, *wnt2*, participates in synapse development. Mutations in *wnt2* result in increased branching of NMJ axons, loss of synaptobrevin, and a 50% reduction in evoked release. Rescue and RNAi data indicates that *wnt2* may function in the postsynaptic muscle. We postulate that Wnt2 may serve as a postsynaptic signal that regulates the development of the presynaptic neuron.

## Results

### *wnt2* Negatively Regulates Synaptic Growth and Alters the Synaptic Distribution of Brp

We previously found that Wnt5 signals via Drl to promote synaptic growth at the *Drosophila* NMJ [29]. Further, *Drosophila* Wg affects synapses both presynaptically by regulating growth of the NMJ [27] and postsynaptically by acting on Fz2 [26]. To investigate whether *wnt2* is involved in synapse development, we examined the 6/7 NMJ of *wnt2^O^* mutant 3^rd^ instar larvae. The *wnt2^O^* mutation introduces a stop codon at residue Q40 likely producing a null mutant. These mutants are viable but male sterile as previously described [43] and exhibit defects in the direct flight muscles due to the requirement of *wnt2* in muscle patterning during pupation [44]. Using qRT-PCR, we observed that *wnt2* RNA is expressed in the larval ventral body wall muscles (ΔC(t) = 9.63±0.24 cycles, n = 6). Careful examination of the *wnt2^O^* mutant larvae showed no visible defects in either the patterning or size of the muscles (Fig. S1: *WT* = 26180±1987 µm^2^, n = 6, *wnt2^O^* = 27130±1473 µm^2^, n = 8, p = 0.70). It's possible that, like other *Wnts*, *wnt2* may function in the larval ventral body wall muscles to regulate synapse development.

**Figure 1:**
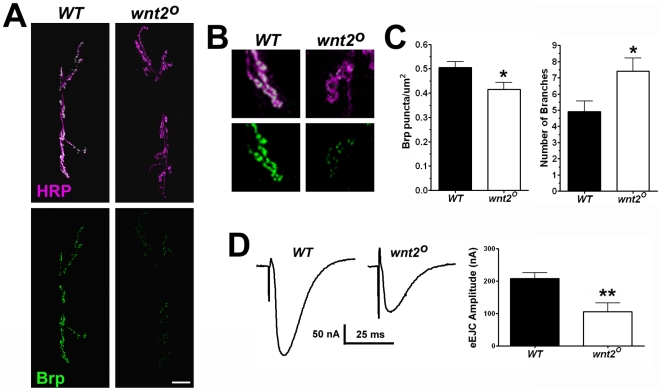
*wnt2* Regulates Synaptic Growth at the Drosophila NMJ and Organization of the Presynaptic Active Zone. A: Confocal micrographs of third instar larval NMJs on ventral longitudinal muscles 6 and 7 immunolabeled with α-HRP (magenta), which labels neuronal membranes, and α-Brp (green). Scale bar  = 20 µm. B: High magnification images of NMJ terminals shown in A. C: Quantification of Brp density (Brp puncta/µm^2^) and the number of branches per NMJ. D: Representative samples of evoked endplate jucntional currents (eEJCs) recorded from muscle 6, which was voltage clamped at −60 mV. (Right) Quantification of EJC amplitudes.

To examine NMJ morphology, we visualized presynaptic motor neurons with the anti-HRP antibody to label neuronal membranes. Our analysis revealed that *wnt2* mutants exhibited an increase in the number of branches compared with controls (Fig. 1A, C; *WT* = 4.92±0.65 branches, n = 12; *wnt2^O^* = 7.40±0.82 branches, n = 10, p = 0.03). Surprisingly, this increase in the number of branches did not produce an increase in the number of boutons (*WT* = 55.85±3.06 boutons, n = 13; *wnt2^O^* = 58.58±3.38 boutons, n = 12, p = 0.55). The size of the presynaptic motor neuron in *wnt2* mutants, however, was approximately 44% larger than controls (*WT* = 348.4±58.89 µm^2^, n = 7; *wnt2^O^* = 501.8±37.78 µm^2^, n = 6, p = 0.04). These data suggest that *wnt2* negatively regulates growth of the presynaptic motor neuron.

We examined the synaptic localization of the presynaptic protein, Bruchpilot/nc82 (Brp) by immunolabeling. Brp is a coiled-coil domain protein that clusters presynaptic Ca^2+^ channels and promotes AZ assembly [10], [45]. The total number of Brp puncta per NMJ was not significantly different in *wnt2* mutants compared with controls (*WT* = 178.4±26.32 Brp puncta per NMJ, n = 7; *wnt2^O^* = 183.8±11.91 Brp puncta per NMJ, n = 8, p = 0.85). Since the amount of Brp at active zones is variable [46], [47], we quantified the mean fluorescence of Brp at the NMJ and found there was a 32% reduction in Brp immunofluorscence in *wnt2* mutants (*WT* = 1.00±0.05 a.u., n = 7; *wnt2^O^* = 0.68±0.09 a.u., n = 7, p = 0.008). We also examined the density of Brp puncta, which is calculated by dividing the total Brp puncta by the area of the presynaptic motor neuron, and found that the density of Brp is slightly but significantly reduced in *wnt2* mutants compared with controls (Fig. 1B, C; *WT* = 0.51±0.02 puncta/µm^2^, n = 9; *wnt2^O^* = 0.42±0.03 puncta/µm^2^, n = 8, p = 0.03) Our data suggests that, although the total number of Brp puncta are similar in *wnt2* mutants and controls, the Brp puncta are more dispersed at the NMJ and may contain less Brp.

To verify that *wnt2* is responsible for the observed phenotype, we examined the NMJ of *wnt2^O^/Df(2R)BSC29* and *wnt2^I^* [43] mutant animals. The number of NMJ branches (*WT* = 4.92±0.65 branches, n = 12; *wnt2^O^* = 7.40±0.82 branches, n = 10, p = 0.03; *wnt2^O^/Df(2R)BSC29* = 7.75±0.53 branches, n = 8, p = 0.006; *wnt2^I^* = 7.83±1.01 branches, n = 6, p = 0.01), density of Brp clusters (*WT* = 0.51±0.02 puncta/µm^2^, n = 9; *wnt2^O^* = 0.42±0.03 puncta/µm^2^, n = 8, p = 0.03; *wnt2^O^/Df(2R)BSC29* = 0.38±0.03 puncta/µm^2^, n = 8, p = 0.01; *wnt2^I^* = 0.39±0.03 puncta/µm^2^, n = 6, p = 0.02), and size of the presynaptic motor neuron (*WT* = 348.4±58.89 µm^2^, n = 7; *wnt2^O^* = 501.8±37.78 µm^2^, n = 6, p = 0.04; *wnt2^O^/Df(2R)BSC29* = 520.1 0±40.25 µm^2^, n = 8, p = 0.03; *wnt2^I^* = 539.0±45.19 µm^2^, n = 6, p = 0.03) exhibited phenotypes similar to the homozygous *wnt2^O^* mutant. These data confirm that *wnt2* is involved in negatively regulating growth of the presynaptic motor neuron and promotes the synaptic localization of Brp.

To determine whether the reduction in the density of Brp affected synaptic function in *wnt2* mutant animals, we recorded ionic currents from postsynaptic muscle. Muscle 6 was voltage clamped at −60 mV and the presynaptic segmental nerve was stimulated (1 Hz, 5 V) to induce synaptic activity. The amplitude of evoked excitatory junctional currents (EJCs) was reduced approximately 50% in *wnt2* mutant animals (Fig. 1D, *WT* = 207.2±18.51 nA, n = 8; *wnt2^O^* = 105.3±26.76 nA, n = 7, p = 0.007). Interestingly, neither the frequency nor amplitude of spontaneous miniature excitatory junctional currents (mEJCs) was significantly different than controls (mEJC frequency: *WT* = 2.10±0.28 Hz, n = 10; *wnt2^O^* = 1.82±0.39 Hz, n = 8, p = 0.56; mEJC amplitude: *WT* = 0.94±0.09 nA, n = 10; *wnt2^O^* = 0.84±0.07 nA, n = 8, p = 0.39). These data, coupled with the altered NMJ morphology, indicates that *wnt2* directly or indirectly regulates both synaptic structure and function.

### Mutations in *wnt2* Preferentially Affect Presynaptic Proteins

**Figure 2:**
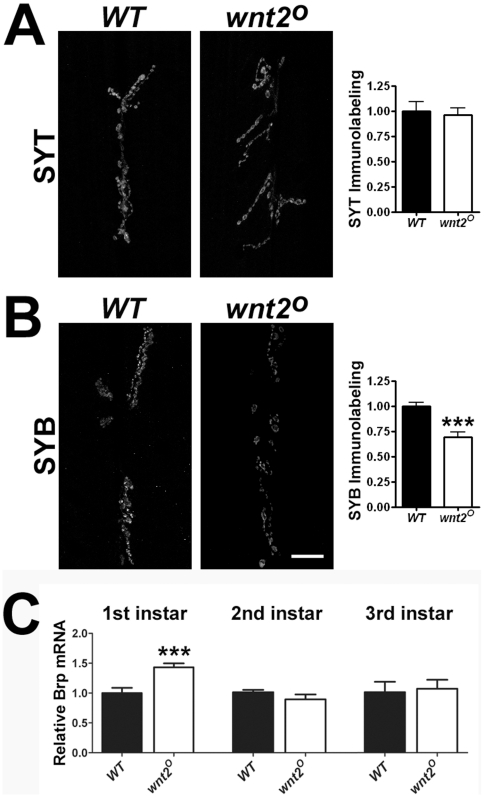
Mutations in *wnt2* Affect Other Presynaptic Proteins. A–B: Confocal images of third instar 6/7 NMJs immunolabeled with α-synaptotagmin (Syt, A) and α-synaptobrevin (Syb, B)with quantification of fluorescence (right histograms). Scale bar  = 20 µm. C: Relative Brp mRNA levels in 1^st^ instar (left), 2^nd^ instar (middle), and 3^rd^ instar (right) larvae, measured by quantitative RT-PCR.

Our electrophysiological results suggest that spontaneous activity is unaffected in *wnt2* mutants. When presented with an evoked stimulus, however, *wnt2* mutants exhibit a large reduction in evoked amplitudes. Evoked release requires the binding of Ca^2+^ to the presynaptic protein synaptotagmin I (Syt) (for reviews see [48], [49]). Therefore, we examined the immunolabeling of Syt and found there was no difference in Syt levels in *wnt2* mutants (Fig. 2A; *WT* = 1.00±0.09 arbitrary units (a.u.), n = 7; *wnt2^O^* = 0.96±0.07 a.u., n = 8, p = 0.75). Similarly, there was no difference in the synaptic vesicle protein, cysteine string protein (CSP, [50]; data not shown, *WT* = 1.00±0.09 a.u., n = 8; *wnt2^O^* = 0.86±0.12 a.u., n = 7, p = 0.36). There was, however, a significant difference in immunolabeling for the presynaptic protein Synaptobrevin (Fig. 2B; Syb: *WT* = 1.00±0.04 a.u., n = 10; *wnt2^O^* = 0.69±0.05 a.u., n = 7, p = 0.0002), which mediates vesicle fusion [51].

We also quantified immunoreactivity for the postsynaptic proteins discs large (DLG) and glutamate receptor subunit IIA (GluRIIA). There was no difference in immunoreactivity of either DLG or GluRIIA (; DLG: *WT* = 1.00±0.06 a.u., n = 8; *wnt2^O^* = 0.95±0.08 a.u., n = 7, p = 0.63; GluRIIA: *WT* = 1.00±0.09 a.u., n = 10; *wnt2^O^* = 1.02±0.10 a.u., n = 9, p = 0.87). Further, there was no detectable immunocytochemical difference in the levels of the cell adhesion molecule FasII and the cytoskeletal component acetylated tubulin (FasII: *WT* = 1.00±0.05 a.u., n = 8; *wnt2^O^* = 0.96±0.10 a.u., n = 8, p = 0.71; synaptic acetylated tubulin, Fig. S1: *WT* = 1.00±0.07 a.u., n = 12; *wnt2^O^* = 0.89±0.08 a.u., n = 11, p = 0.31; muscle 6 acetylated tubulin: *WT* = 1.00±0.10 a.u., n = 12; *wnt2^O^* = 0.99±0.06 a.u., n = 11, p = 0.93). Taken together, our electrophysiological and immunocytochemical results suggest that *wnt2* is involved in the expression or localization of presynaptic proteins.

### The Loss of Brp and Syb is not Due to Transcriptional Mechanisms

**Figure 3:**
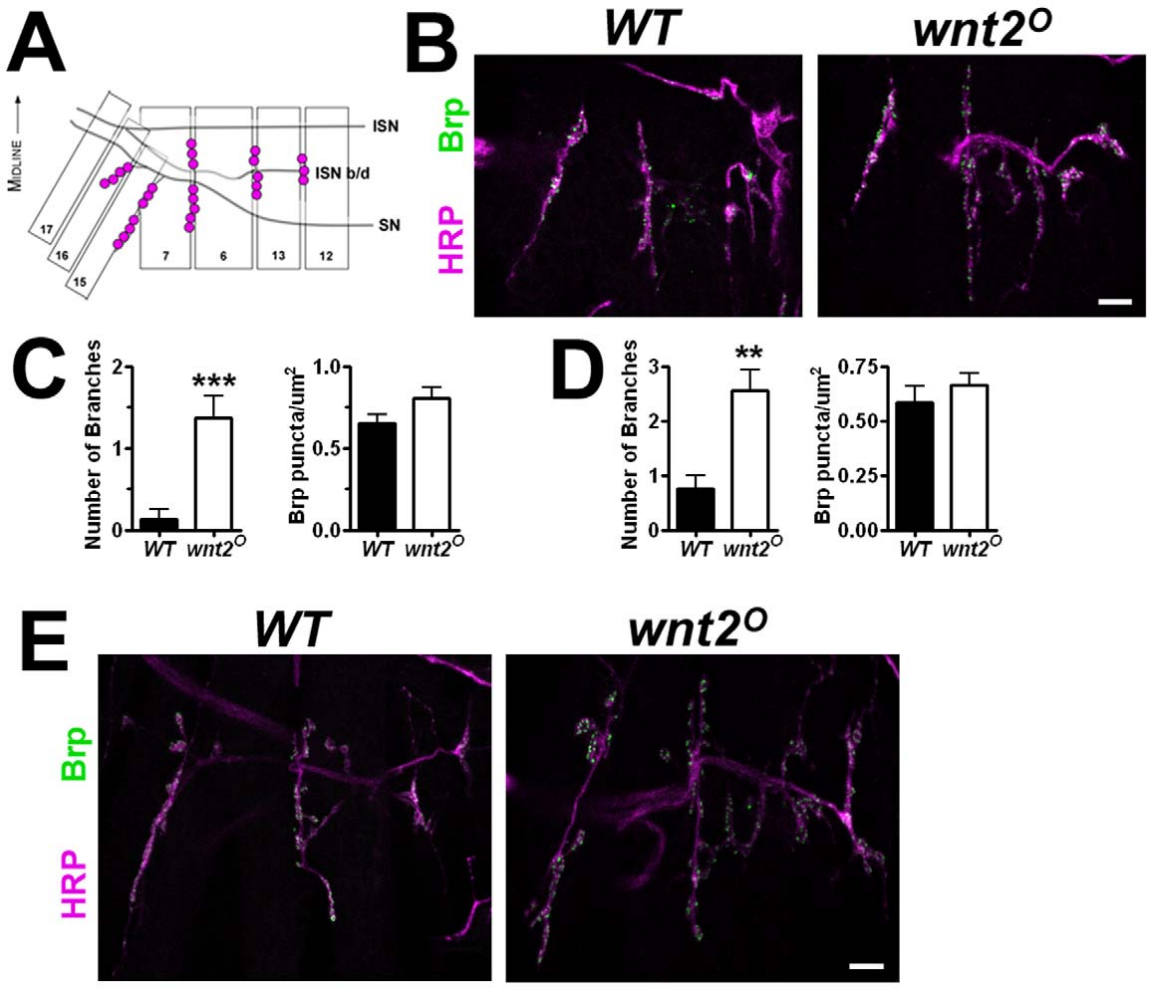
The Morphological Change in *wnt2* Mutants Occurs Early in Development. A: Schematic of the 6/7 and neighboring NMJs in pre-3^rd^ instar larvae. B: Confocal fluorescent images showing 1^st^ instar larval NMJs of *WT* and *wnt2^O^* mutants labeled with antibodies against HRP (magenta) and Brp (green). Scale bar  = 5 µm. C: Quantification of the number of branches per NMJ (left) and Brp density (right) in 1^st^ instar larvae.D: Quantification of the number of branches per NMJ (left) and Brp density (right) in 2^nd^ instar larvae. E: Confocal fluorescent images showing 2^nd^ instar larval NMJs labeled with antibodies against HRP (magenta) and nc82 (green). Scale bar: 5 µm.

The binding of Wnt ligands to their receptors can activate downstream pathways that lead to transcription by the TCF/LEF family of transcription factors [52]. To determine if reduced Brp density and loss of Syb in *wnt2* mutants was due to changes in gene transcription, we examined *brp* and *syb* mRNA levels in 1^st^, 2^nd^, and 3^rd^ instar larvae using quantitative RT-PCR. Surprisingly, there was a significant increase in *brp* mRNA in 1^st^ instar larvae (Fig. 2C; *WT* = 1.00±0.09 a.u., n = 6; *wnt2^O^* = 1.43±0.07 a.u., n = 6, p = 0.003) consistent with the slight increase in Brp density in 1^st^ instar larvae (see Fig. 3). However, there was no significant difference in *brp* mRNA levels in either 2^nd^ or 3^rd^ instar larvae (Fig. 2D; *WT* 2^nd^ instar = 1.00±0.04 a.u., n = 6; *wnt2^O^* 2^nd^ instar = 0.88±0.08 a.u., n = 5, p = 0.20; *WT* 3^rd^ instar = 1.00±0.17 a.u., n = 6; *wnt2^O^* 3^rd^ instar = 1.06±0.15 a.u., n = 6, p = 0.81). Nor was there any significant difference in *syb* mRNA levels between controls and *wnt2* mutants at any developmental stage examined (*WT* 1^st^ instar = 1.00±0.05 a.u., n = 6; *wnt2^O^* 1^st^ instar = 0.94±0.07 a.u., n = 6, p = 0.50; *WT* 2^nd^ instar = 1.00±0.10 a.u., n = 6; *wnt2^O^* 2^nd^ instar = 0.98±0.12 a.u., n = 5, p = 0.88; *WT* 3^rd^ instar = 1.00±0.27 a.u., n = 6; *wnt2^O^* 3^rd^ instar = 1.43±0.39 a.u., n = 6, p = 0.39). These data indicate that reduced density of Brp and loss of Syb at the NMJ of *wnt2* mutants is not due to changes in *brp* and *syb* transcript levels.

### The Morphological Change in *wnt2* Mutants Occurs Early in Development

Growth of the NMJ during larval development (24–120 h AEL) involves the addition of new synaptic boutons, branches, and AZs per bouton [53]–[55]. The dramatic increase in presynaptic growth attempts to accommodate the rapidly growing larval muscles and requires transsynaptic signaling between cells (for review see [53]). To ascertain whether the increase in branch number and reduction in AZs resulted from a failure to coordinate growth of the presynaptic motor neuron and postsynaptic muscle, we examined *wnt2* mutant NMJs during both 1^st^ and 2^nd^ instar larval stages (24 and 48 h AEL, respectively). Figure 3 (A) depicts NMJs on the ventral longitudinal body wall muscles of a single 1^st^ instar hemisegment. The segmental nerve innervates muscles 15, 16, 17, 7, 6, 13, and 12 while the intersegmental nerve innervates muscle 7, 6, 13, and 12. Comparison of *wnt2* mutants to this highly stereotyped pattern revealed a significant increase in branch numbers at the 6/7 NMJ during both the 1^st^ and 2^nd^ instar larval stages (Fig. 3B-E, 1^st^ instar: *WT* = 0.13±0.13 branches, n = 8; *wnt2^O^* = 1.38±0.26 branches, n = 8, p = 0.0007; 2^nd^ instar: *WT* = 0.75±0.25 branches, n = 8; *wnt2^O^* = 2.56±0.38 branches, n = 9, p = 0.0015) with no change in bouton numbers (Fig. 3B-E, 1^st^ instar: *WT* = 11.13±1.09 branches, n = 8; *wnt2^O^* = 12.63±0.80 boutons, n = 8, p = 0.29; 2^nd^ instar: *WT* = 19.44±0.91 boutons, n = 9; *wnt2^O^* = 17.11±1.00 boutons, n = 9, p = 0.11). This is similar to the phenotype observed in 3^rd^ instar mutant animals. Interestingly, in contrast to the reduction in Brp density observed during the 3^rd^ instar larval stage, there was no difference in the density of Brp puncta during the 1^st^ and 2^nd^ instar larval stages (1^st^ instar: *WT* = 0.65±0.05 puncta/µm^2^, n = 8; *wnt2^O^* = 0.81±0.061 puncta/µm^2^, n = 8, p = 0.08; 2^nd^ instar: *WT* = 0.56±0.07 puncta/µm^2^, n = 7; *wnt2^O^* = 0.66±0.06 puncta/µm^2^, n = 9, p = 0.40). These data indicate that the loss of *wnt2* affects the growth of the NMJ early in development but does not affect the density of Brp until later in development. This suggests that different mechanisms may govern NMJ structure and function in the *wnt2* mutant.

### *wnt2* May Function in Postsynaptic Muscle to Mediate Brp Density and Structure of the Presynaptic Neuron

**Figure 4:**
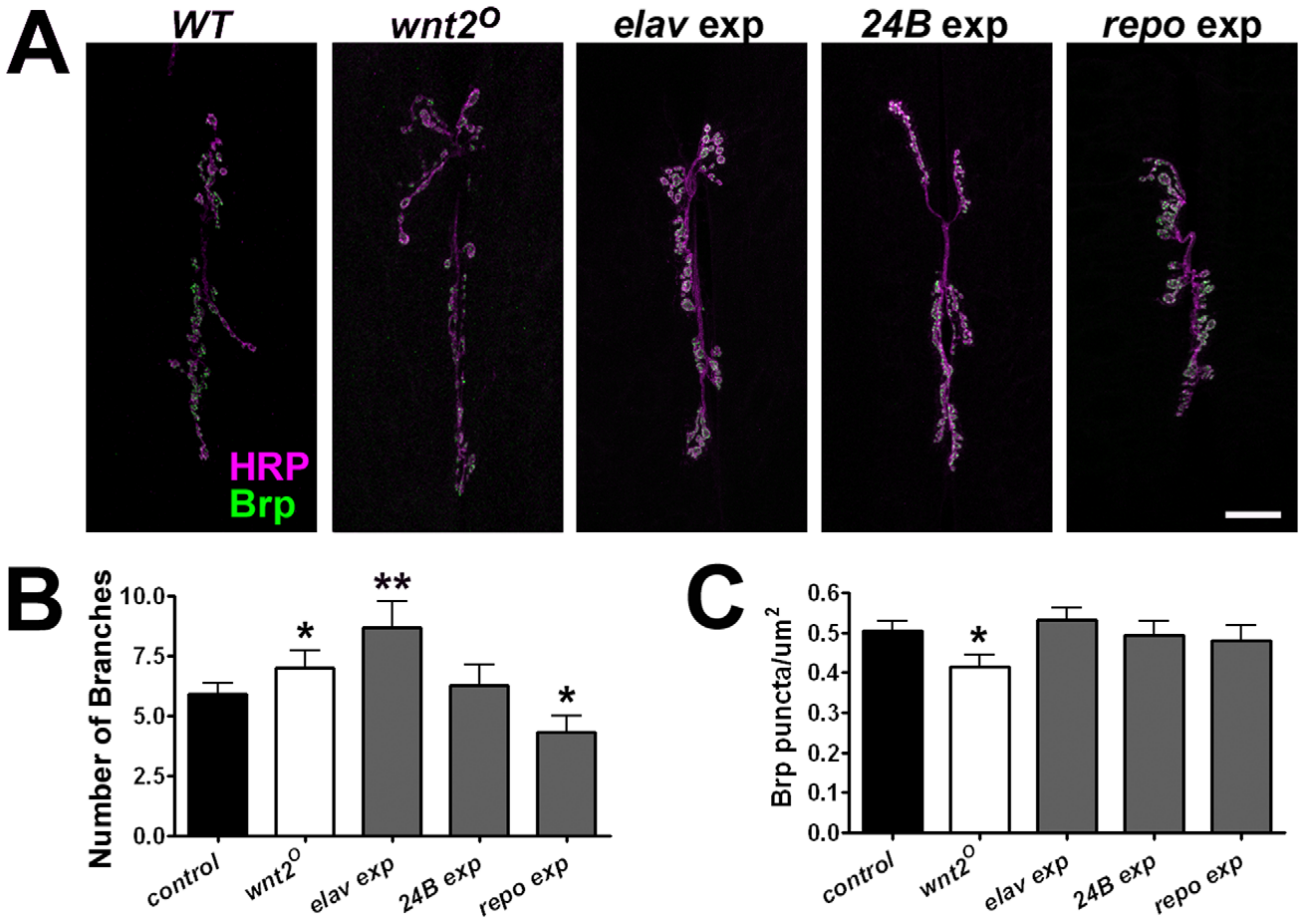
Restoration of *wnt2* in Postsynaptic Muscle Rescues Presynaptic Morphology and Brp density. A: *wnt2* expression was restored in the *wnt2^O^* mutant background using a *UAS-wnt2* transgene driven in either neurons (*elav*), muscle (*24B*) or glial cells (*repo*). Representative confocal micrographs show the 6/7 NMJ immunolabeled with HRP (magenta) to visualize neuronal membranes and Brp (green). Scale bar  = 20 µm. B: Quantification of the number of branches per 6/7 NMJ. C: Quantification of Brp density.

Our attempts to make an antibody that specifically recognized the Wnt2 protein were unsuccessful. Thus, we used genetic techniques to delineate the cell type expressing the *wnt2* gene. First, we performed cell-type specific gene rescue experiments by expressing the *UAS-wnt2* transgene specifically in neurons (using the *elav-Gal4* driver), muscle (using the *24B-Gal4* driver), or glial cells (using the *repo-Gal4* driver) in the *wnt2^O^* mutant background. Expression of *wnt2* in muscle rescued the number of branches to near control levels. Conversely, expression of *wnt2* in neurons of *wnt2^O^* mutants produced a further increase in branch number while expression in glia significantly reduced branch numbers (Fig. 4 A–B; control = 5.88±0.46 branches, n = 16; *elav>wnt2* = 8.67±1.11 branches, n = 9, p = 0.01; *24B>wnt2* = 6.25±0.86 branches, n = 12, p = 0.69; *repo>wnt2* = 4.30±0.67 branches, n = 10, p = 0.047). Expression of *wnt2* in all three cell types rescued Brp density (control = 0.51±0.02 puncta/µm^2^, n = 9; *elav>wnt2* = 0.53±0.03 puncta/µm^2^, n = 9, p = 0.52; *24B>wnt2* = 0.49±0.04 puncta/µm^2^, n = 10, p = 0.82; *repo>wnt2* = 0.48±0.04 puncta/µm^2^, n = 8, p = 0.61) suggesting expression of *wnt2* in any NMJ cell type is sufficient to rescue the localization of Brp.

**Figure 5:**
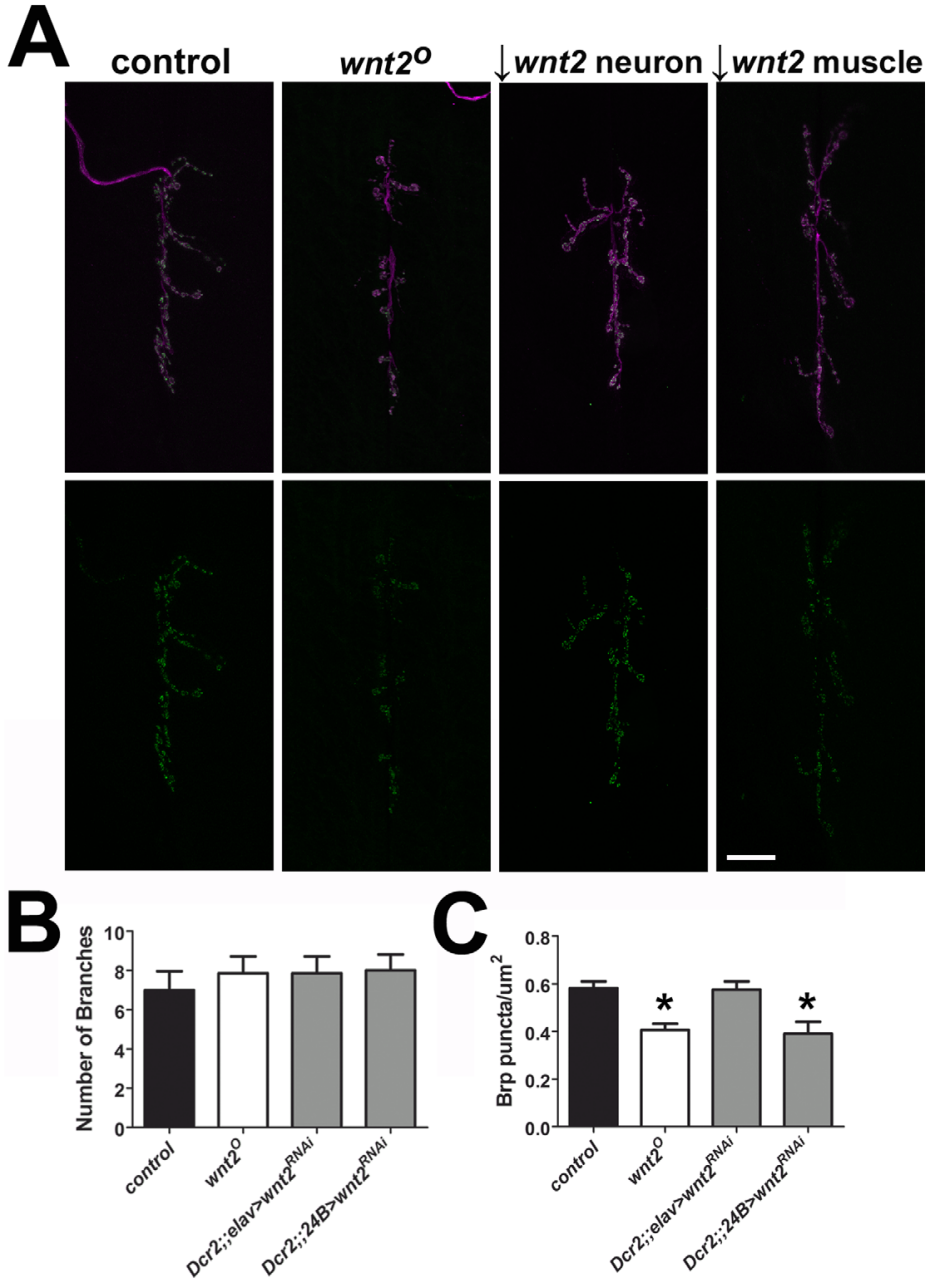
Knockdown of *wnt2* in Postsynaptic Muscle Mimics the *wnt2* Mutant Phenotype. A: *wnt2* function was reduced using a *UAS-wnt2^RNAi^* transgene driven in either motor neurons (*Dcr2;;elav*) or muscle cells (*Dcr2;;24B*). Representative confocal micrographs show the 6/7 NMJ immunolabeled with HRP (magenta) to visualize neuronal membranes and Brp (green). Scale bar  = 20 µm. B: Quantification of the number of branches per 6/7 NMJ. C: Quantification of Brp density.

Since Wnt2 is a secreted protein, genetic rescue could have occurred by expression from extraneous cell types. To verify and further clarify the above results, we knocked down *wnt2* function in neurons or muscle. Expression of a *UAS-wnt2^RNAi^* construct within neurons (using the *Dcr2;;elav* driver) produced no change in either the number of branches or Brp density compared with controls (Fig. 5; branches: *UAS-wnt2^RNAi^*/*+* = 7.43±1.69, n = 7; *Dcr2;;elav* = 8.50±0.89, n = 6; *Dcr2;;elav >UAS-wnt2^RNAi^* = 7.86±0.86, n = 7, p = 0.61: Brp density: *UAS-wnt2^RNAi^*/*+* = 0.63±0.04, n = 7; *Dcr2;;elav* = 0.58±0.03, n = 6; *Dcr2;;elav>UAS-wnt2^RNAi^* = 0.58±0.03, n = 6, p = 0.89). Knockdown of *wnt2* in postsynaptic muscle, however, produced a Brp phenotype similar to that of the *wnt2^O^* mutant (Fig. 5; branches: *UAS-wnt2^RNAi^*/*+* = 7.43±1.69, n = 7;; *Dcr2;;24B* = 6.83±1.05 n = 6; *Dcr2;;24B>UAS-wnt2^RNAi^* = 8.00±0.82, n = 7, p = 0.39: Brp density: *UAS-wnt2^RNAi^*/*+*  = 0.63±0.04, n = 7; *Dcr2;;24B* = 0.56±0.05, n = 6; *24B>UAS-wnt2^RNAi^* = 0.39±0.05, n = 7, p = 0.04). To verify the RNAi transgenes reduced the expression of *wnt2* and did not affect the levels of *brp* transcript, we performed qRT-PCR for *wnt2* and *brp* (*wnt2*: control = 1.00±0.04 a.u., n = 6; *Dcr2;;elav>UAS-wnt2^RNAi^* = 0.79±0.01 a.u., n = 6, p = 0.003; *Dcr2;;24B>UAS-wnt2^RNAi^* = 0.76±0.01 a.u., n = 6, p<0.0001; *brp*: control = 1.00±0.04 a.u., n = 6; *Dcr2;;24B>UAS-wnt2^RNAi^* = 0.9914±0.027 a.u., n = 6, p = 0.73). Our rescue and RNAi data collectively indicate that *wnt2* functions in the postsynaptic muscle to regulate the density of Brp.

### Wnt2 does not Likely Exert its Presynaptic Effects via Drl or Fz Receptors

**Figure 6:**
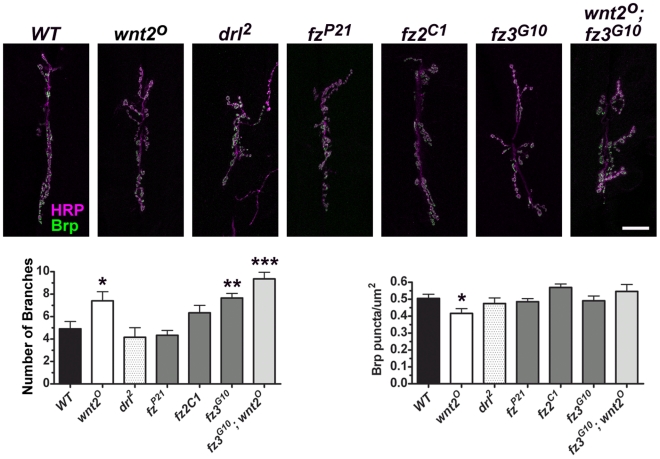
The Effects of Wnt2 are not likely mediated by Drl or Fz Receptors. Top panels: Representative confocal micrographs show the 6/7 NMJ immunolabeled with HRP (magenta) to visualize neuronal membranes and Brp (green). Scale bar  = 20 µm. Bottom left: Quantification of the number of branches per 6/7 NMJ. Bottom right: Quantification of Brp density.

Previous experiments have demonstrated that Wingless utilizes postsynaptic DFz2 receptors to influence *Drosophila* NMJ morphology [26], [56], [57]. We sought to determine the receptor through which Wnt2 mediated its synaptic effects by examining mutant NMJs of *fz* and *drl* mutants (Figure 6). Previous data indicates Wnt2 binds to Fz, Fz2, and Fz3 with similar affinities [58]. If Wnt2 signals via a single receptor or a combination of receptors, then mutants for those receptors should phenocopy *wnt2* mutants. *fz3^G10^* null mutant animals [59] most closely resembled the *wnt2^O^* NMJ phenotype in terms of morphology (Fig. 6; *WT* = 4.92±0.66 branches, n = 12; *wnt2^O^* = 7.40±0.82 branches, n = 10, p = 0.025; *drl^2^* = 4.17±0.84 branches, n = 12, p = 0.49; *fz^D21^* = 4.33±0.44, n = 9, p = 0.50; *fz2^C1^* = 6.29±0.57, n = 7, p = 0.17; *fz3^G10^* = 7.67±0.41, n = 9, p = 0.004). None of the mutants examined exhibited a significant change in Brp density (Fig. 6; *WT* = 0.51±0.02 puncta/µm^2^, n = 9; *wnt2^O^* = 0.42±0.03 puncta/µm^2^, n = 8, p = 0.03; *drl^2^* = 0.47±0.03 puncta/µm^2^, n = 8, p = 0.46; *fz^P21^* = 0.48±0.02 puncta/µm^2^, n = 8, p = 0.53; *fz2^C1^* = 0.53±0.03, n = 7 puncta/µm^2^, p = 0.59; *fz3^G10^* = 0.49±0.03 puncta/µm^2^, n = 9, p = 0.72).

The increase in NMJ branch numbers in both *wnt2* and *fz3* mutants raised the possibility that the two genes may function in the same signaling pathway to regulate synaptic growth. To test this possibility, we constructed double mutants bearing simultaneous *wnt2* and *fz3* mutations. The NMJ morphology of *wnt2^O^; fz3^G10^* double mutants was statistically different than *WT* and of each of the single mutants (Fig. 6, *wnt2^O^* = 7.40±0.82 branches, n = 10; *fz3^G10^* = 7.67±0.41, n = 9, p = 0.004; *wnt2^O^; fz3^G10^* = 9.36±0.59, n = 11, p = 0.04) suggesting that *wnt2* and *fz3* may each regulate synaptic growth independently of the other. Collectively, our data suggest that Wnt2 is expressed by the postsynaptic muscle and is involved in presynaptic protein localization.

## Discussion

Synapse development is a complex process that requires pre- and postsynaptic cells to maintain constant communication with one another via transsynaptic signaling. Molecules with well established roles in this process include cell adhesion molecules [60], Ephrin ligands and Eph receptors [61], and the classical cadherins [62]. We provide evidence that Wnt2 may act as a signaling molecule that is expressed by the postsynaptic muscle where it acts on the presynaptic cell to directly or indirectly regulate size of the presynaptic motor neuron and promote protein localization.

### Wnt2 Regulates NMJ Development and Localization of Presynaptic Proteins

We present several pieces of evidence to support our conclusion that Wnt2 regulates development of the NMJ. *wnt2* mutations produce overgrown NMJs with an increased number of branches (Fig. 1). The significant increase in NMJ branches is present early in development as both 1^st^ and 2^nd^ instar mutant larvae also exhibit an overgrowth (Fig. 3). This could indicate that *wnt2* is required shortly after synapse formation to regulate NMJ growth. Although the NMJ is enlarged in the *wnt2* mutant, the number of Brp puncta remained similar to controls. The level of Brp immunfluorescence is reduced, however, suggesting that the amount of Brp protein per punctum is decreased. Since Brp is localized to active zones where it promotes Ca^2+^ channel clustering [63], reduced staining of Brp puncta may indicate that functioning of the active zones are compromised. A recent paper however, reported that the majority of active zones in *Drosophila rab3* mutants do not contain Brp [64].

Electrophysiological recordings from muscle 6 of *wnt2* mutants showed that the amplitudes of evoked events were significantly reduced without a reduction in the frequency or amplitude of spontaneous events (Fig. 1). This intriguing finding led us to carefully examine the concentrations of presynaptic proteins including Syt, Syb, CSP, Brp. The levels of Syb and Brp were significantly reduced in the *wnt2* mutant as indicated by immunocytochemistry (Fig. 2). Syb is a synaptic vesicle associated protein that assembles with syntaxin and SNAP-25 to form the SNARE complex, which renders vesicles competent for fusion (for reviews see [65], [66]). The electrophysiological phenotype we observed in *wnt2* mutants is consistent with both the *syb* and *brp* mutant phenotypes. Syb is required for evoked but not spontaneous transmission in *Drosophila* [67] and knockdown of *brp* in neurons reduces evoked responses while preserving spontaneous transmission [10]. Thus, our finding that the total number of Brp puncta in *wnt2* mutants is unchanged coupled with the significant reduction in evoked responses, suggests that there may be a reduction in the number of functional active zones in the *wnt2* mutant. Indeed, we observed that the immunolabeling of Brp puncta is reduced, suggesting that the amount of Brp protein per puncta is decreased.[51]

The reduced labeling of Brp and Syb in the presynaptic motor neuron of *wnt2* mutants is not likely due to changes in transcriptional mechanisms. Messenger RNA levels of both *brp* and *syb* are similar in mutant and control animals (Fig. 2). It is possible that the observed changes in Brp and Syb are due to mislocalization of mRNA. Another possibility is that the loss of *wnt2* leads to mislocalization of presynaptic proteins. Rat Wnt7a, which is 77.1% similar in amino acid sequence to Wnt7b [68], when applied to hippocampal cultures, induces clustering of Syt, SV2, and increases the number of clusters containing synaptophysin [23]. Both Wnt7a and Wnt7b induce clustering of synapsin I in mouse cerebellar granule cell cultures. Treatment of culture medium with Wnt7b increased Bassoon clustering but did not increase total protein levels as indicated by Western Blots [69].

### Wnt2 is Expressed by Postsynaptic Muscle

Wnts are secreted glycoproteins. An important aspect of understanding the function of Wnt2 is to determine where at the synapse it functions to regulate presynaptic motor neuron morphology and localization of proteins. Our cell-type specific cDNA expression in *wnt2* mutants showed that Wnt2 may function in either presynaptic motor neurons or postsynaptic muscle. Expression in either motor neurons or muscle restored presynaptic Brp density. Expression in postsynaptic muscle also restored NMJ morphology while expression in presynaptic motor neurons caused a further increase in the number of NMJ branches (Fig. 4). Knockdown of *wnt2* in muscle produced a phenotype similar to that of null mutant (Fig. 5). Our results collectively suggest that Wnt2 may be expressed by the postsynaptic muscle where it acts as a retrograde signal that negatively regulates NMJ growth and promotes the localization of presynaptic proteins. Based on our data, we cannot conclude whether *wnt2* directly or indirectly regulates these synaptic characteristics.

A number of other molecules have been implicated in retrograde synaptic signaling including Ankyrin [70], nitric oxide [71], SAP97 [72], Synaptotagmin 4 [73], and secreted proteins such as Glass Bottom Boat [74], fibroblast growth factors [75], and Wnts [69], [76]–[78]. Mouse Wnt3 is secreted from motor neurons where it increases the size of growth cones and branching of incoming sensory neurons [24]. Similarly, mouse Wnt7a is expressed by cerebellar granule cells and acts on presynaptic mossy fibers to remodel axons and growth cones [22]. The receptor(s) that mediate the above effects are, as yet, unidentified but Wnt ligand binding to its receptor induces cytoskeletal changes [79], [80].

We sought to determine the receptor through which Wnt2 signaled by examining mutants for *drl*, *fz*, *fz2*, and *fz3*. None of the mutants exhibited a reduction in the density of Brp. Mutations in *fz3*, however, led to a significant increase in NMJ branches similar to that of the *wnt2* mutant. This raised the possibility that Wnt2 was signaling via Fz3 to negatively regulate NMJ growth. *wnt2^O^; fz3^G10^* double mutants, however, exhibited a significant increase in NMJ branches greater than that of the single mutants (Fig. 6) suggesting *wnt2* and *fz3* act independently of one another to regulate synaptic growth. Wnt2 may signal via the Wnt receptors Fz4 or Smo but binding assays indicate there is no detectible binding between Wnt2 and these receptors [58]. It is also possible that we did not detect a phenotype in Frizzled mutants due to functional redundancy of these receptors. Future work will be required to uncover the receptor that mediates Wnt2 signaling.

## Materials and Methods

### Fly Stocks

All animals were raised at 25°C in standard fly vials with corn meal molasses medium. Wnt2 fly stocks along with the all *Gal4* lines were obtained from Bloomington *Drosophila* Stock Center. *fz^D21^*, *fz2^C1^*, *fz3^G10^*, and *drl^2^* fly stocks were generous gifts from the labs of Roel Nusse, Gary Struhl, Kaoru Saigo, and John Thomas, respectively. The *UAS-wnt2^RNAi^* line was provided by the Vienna *Drosophila* RNAi Center (v38079).

### Antibodies and Immunocytochemistry

For staining and microscopy, animals were dissected and fixed for 30–60 min in either Bouin's fixative (when GluRIIA or nc82 antibodies were used), or 4% paraformaldehyde (for all other immunolabeling). First and second instar larvae were dissected and fillet preparations were glued down using Sylgard-coated coverslips. Third instar larvae were dissected and fillet preparations were pinned down in Sylgard lined Petri dishes. All dissections were done in *Drosophila* standard saline (135 mM NaCl, 5 mM KCl, 4 mM MgCl, 1.8 mM CaCl, 5 mM TES, 72 mM sucrose) with 2 mM glutamate to preserve neuronal morphology [81] at RT.

The following mouse monoclonal antibodies from the Iowa Developmental Hybridoma Bank (Iowa City, IA) were used: anti-Brp (nc82, 1∶50), anti-DLG (1∶1000), anti-GluRIIA (1∶100), anti-FasII (1∶200), anti-CSP (1∶200), and anti-Syt (1∶200). Rabbit polyclonal anti-Syb (1∶1000) was a gift from Hugo Bellen. Mouse monoclonal anti-acetylated tubulin (Sigma) was used at 1∶1000. F-actin was labeled using rhodamine-conjugated phallotoxin at 1∶200 (Invitrogen, Carlsbad, CA). Fluorescently conjugated anti-HRP (Jackson Immunoresearch Labs, West Grove, PA) was used at 1∶100. Goat anti-rabbit or goat anti-mouse fluorescent (FITC or TRITC) secondary antibodies (Jackson Immunoresearch Labs, West Grove, PA) were used at 1∶400. The 6/7 NMJ of abdominal hemisegments A3 or A4 were used for all studies. Confocal images were obtained using a Zeiss LSM 510 laser-scanning confocal microscope. Figure 5 and Figure S1 images were obtained using an Olympus FV1000 laser-scanning confocal microscope. Image analysis and quantification was performed using ImageJ and Adobe Photoshop software.

### Electrophysiology

All electrophysiology was performed on ventral body wall muscle 6. Larval recordings were performed on third instar larvae 110–120 hr AEL. Muscle 6 was voltage-clamped at −60 mV. Standard two-electrode voltage clamp techniques were used, as previously described [82]. Data were acquired and analyzed using an Axopatch amplifier and pClamp9 (Axon Instruments, Union City, CA). All dissections and recordings were done in standard *Drosophila* saline at 19C.

### qRT-PCR

*wnt2^O^* and control animals were homogenized and RNA was extracted using TRIreagent (Sigma, St. Loius, MO). RNA was obtained from 1^st^ instar (24–26 h after egg laying (AEL)), 2^nd^ instar (48–50 h AEL), and 3^rd^ instar larvae (110–120 h AEL). Reverse transcription of RNA was performed using Qiagen's Quantifast Sybr Green RT-PCR kit (Valencia, CA) with mRNA specific primers for actin (forward primer: GCACCACACCTTCTACAATGAGC, reverse primer: TACAGCGAGAGCACAGCCTGGATG), Brp (forward primer: GCAAGAGGATTAAACGAACGAG, reverse primer: TAGCGGGTTCTTGGATAGTC), Syb (forward primer: GCACATTGTCAAGCAAATTCAC, reverse primer: TGTTGTTCCTGATTTGATGGTC), and Wnt2 (forward primer: ATTGTGGAACTGTGGAACTG, reverse primer: GCTGGACACTAATCTTATTTCC). Primers were designed against exon-intron borders and no primer sets yielded products when DNA was used as a template. qRT-PCR reactions were run using a Stratagene MX3000P. To obtain ΔC(t) values, the cycle threshold (C(t)) for Brp, Syb, and Wnt2 along with an actin control were measured for each sample. The difference between the mRNA-specific primer and the actin C(t) were calculated to determine the ΔC(t) value. ΔC(t) values were normalized to *WT* by dividing each ΔC(t) value by the mean ΔC(t) value for *WT* to yield the mRNA levels relative to *WT*. There was no significant differences in the actin C(t) values between *WT* and *wnt2* mutants. For the qRT-PCR experiments examining *wnt2* expression in nervous system and ventral body wall muscles, nervous systems and ventral body wall muscles were extracted from third instar larvae. The brain, ventral nerve cord, and intersegmental nerves were first extracted followed by the ventral body wall muscles. To minimize contamination of other tissue types in the ventral body wall muscles, only ventral body wall muscles between A3-A5 were used for RNA extraction.

### Data Acquisition and Statistics

The total number of boutons and branches were acquired from 6/7 NMJs of hemisegments A3 or A4 of all animals. Branches were defined as an extension of the presynaptic motor neuron that included more than one bouton. The density of Brp labeling was quantified by counting the total number of Brp puncta in a projected Z-image and dividing by the total NMJ area as indicated by Brp labeling using ImageJ (NIH) software. We quantified immunoreactivity for all other synaptic proteins by measuring the mean fluorescence intensity of the NMJ using Adobe Photoshop software and subtracting the mean non-NMJ background over an identical area of the neighboring muscle membrane. For DLG and muscle acetylated tubulin, the average background from a non-synaptic, non-muscle area was used.

Statistics were performed using GraphPad Prism (v. 4.01., 5.01). All statistical comparisons were made using unpaired students t-tests. Unless otherwise noted, control animals used were *WT-Berlin*, *elav-Gal4*,*24B-Gal4, wnt2^O^;elav/+, wnt2^O^;24B/+, and wnt2^O^; UAS-wnt2*. There was no significant difference in bouton or branch numbers or Brp puncta density between genotypes. Therefore, the data were combined into one control group where appropriate. Statistical significance in figures is represented as follows: * = p<0.05, ** = p<0.01, and *** = p<0.001. All error bars represent S.E.M.

## Supporting Information

Figure S1 Muscle size in wnt2 mutants is similar to that of controls. A: Representative confocal micrographs show the 6/7 NMJ labeled with HRP (green) to visualize neuronal membranes and phallotoxin to label F-actin (magenta). Scale bar  = 20 µm. B: Quantification of muscle sizes in controls and wnt2 mutants. C: Representative confocal micrographs show the 6/7 NMJ immunolabeled with HRP (magenta) and acetylated tubulin (green). Scale bar  = 20 µm.
(0.72 MB TIF)
