# Supplementary figures and images for: Mutations in Wnt2 Alter Presynaptic Motor Neuron Morphology and Presynaptic Protein Localization at the Drosophila Neuromuscular Junction
Source: PLoS One. 2010 Sep 15;5(9):e12778. doi: 10.1371/journal.pone.0012778 (PMC2939895; doi:10.1371/journal.pone.0012778)

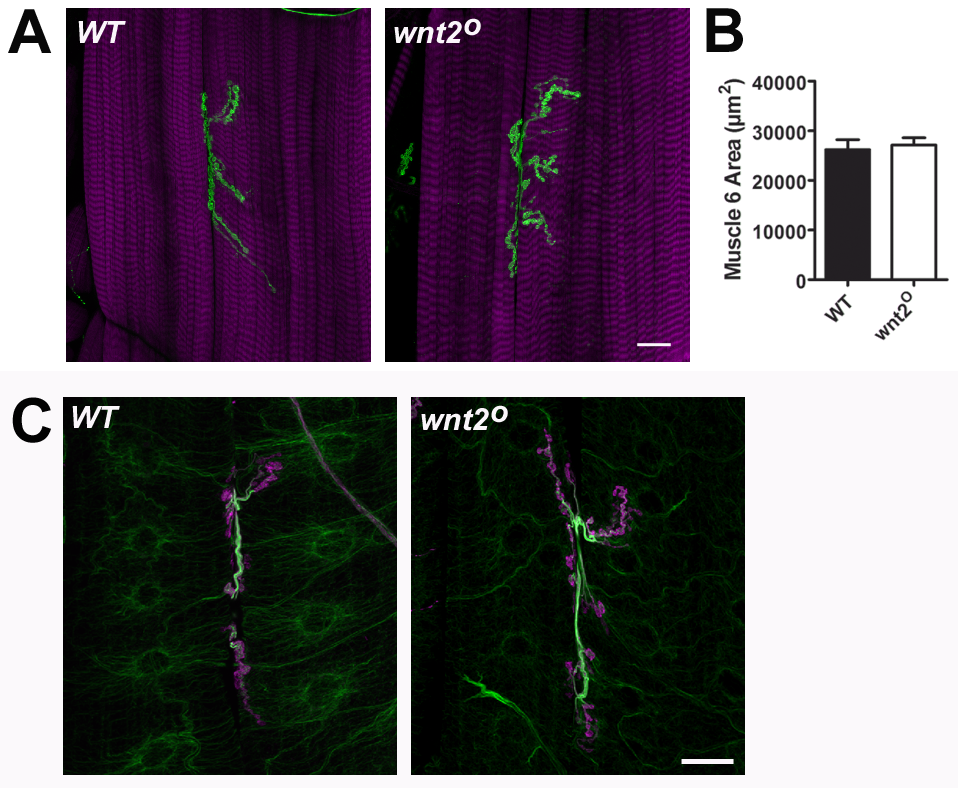

Supplement: Figure S1 — Muscle size in wnt2 mutants is similar to that of controls. A: Representative confocal micrographs show the 6/7 NMJ labeled with HRP (green) to visualize neuronal membranes and phallotoxin to label F-actin (magenta). Scale bar = 20 µm. B: Quantification of muscle sizes in controls and wnt2 mutants. C: Representative confocal micrographs show the 6/7 NMJ immunolabeled with HRP (magenta) and acetylated tubulin (green). Scale bar = 20 µm. (0.72 MB TIF) [file pone.0012778.s001.tif]
